# Serum Uric Acid as an Independent Risk Factor for the Presence and Severity of Early-Onset Coronary Artery Disease: A Case-Control Study

**DOI:** 10.1155/2018/1236837

**Published:** 2018-10-23

**Authors:** Ting-Ting Tian, Hui Li, Sheng-Jie Chen, Qing Wang, Qing-Wu Tian, Bei-Bei Zhang, Jie Zhu, Guo-Wei He, Li-Min Lun, Chao Xuan

**Affiliations:** ^1^Department of Clinical Laboratory, The Affiliated Hospital of Qingdao University, Qingdao, China; ^2^Department of Molecular Microbiology, Oslo University Hospital, Oslo, Norway; ^3^Department Scientific Research Management, The Affiliated Hospital of Qingdao University, Qingdao, China; ^4^Department of Cardiovascular Surgery, TEDA International Cardiovascular Hospital, Academy of Medical Sciences & Peking Union Medical College, Tianjin, China; ^5^Department of Surgery, Oregon Health and Science University, Portland, Oregon, USA

## Abstract

Serum uric acid (UA) is the final product of purine metabolism in humans. The present study is aimed at identifying the potential association between serum UA and early-onset coronary artery disease (EOCAD). The study population consisted of 1093 EOCAD patients aged ≤50 years, and 1117 age- and sex-matched apparently healthy people served as controls. The concentrations of UA were measured by uricase method. The severity of CAD was evaluated by Gensini score. The mean serum level of UA was 5.843 ± 1.479 mg/dl in EOCAD patients and 5.433 ± 1.529 mg/dl in controls. Serum UA levels were significantly higher in the EOCAD group than those in the control group (*P* < 0.001) and was an independent risk factor for EOCAD (OR = 1.100, 95% CI: 1.022–1.185). The early-onset myocardial infarction patients with 3-vessel disease had higher serum UA levels than those with 1- or 2-vessel disease. The serum UA levels of EOCAD patients with acute coronary syndrome were significantly higher than those with chronic coronary artery disease. EOCAD patients with hyperuricemia had higher Gensini scores than those without hyperuricemia. In addition, the serum UA levels were affected by drinking (*P* < 0.01) and were positively correlated with serum creatinine (*r* = 0.323) and weight (*r* = 0.327). Our results show that serum UA was an independent risk factor for EOCAD. The serum UA levels were associated with the presence and severity of EOCAD and suggested that UA may be involved in the progression of EOCAD.

## 1. Introduction

Uric acid (UA) is the end product of purine nucleotide metabolism, formed from the breakdown of adenosine and guanine [1]. UA is a weak acid with a pH of 5.8. UA mainly exists as urate, the salt of UA, and is excreted in urine [2]. The solubility of UA in human blood is low. When the serum level of UA is higher than the solubility limit (6.8 mg/dl), crystals of UA form as monosodium urate [3].

Nitric oxide (NO) plays important roles in the regulation of vascular tone and structure [4]. Decreasing in the production and the bioavailability of NO is an important symbol of endothelial dysfunction [5]. UA is involved in the reduction of NO bioavailability via a variety of mechanisms in endothelial cells, including blocking uptake of L-arginine, stimulating L-arginine degradation, and by scavenging of NO from UA-generated oxidants or by UA itself [6]. It has been reported that UA inhibits the NO-dependent dilation of isolated aortic rings in rats [7]. In addition, it is well known that crystal UA can induce an inflammatory response [8], and soluble uric acid has been demonstrated to induce vascular smooth muscle cell proliferation in vitro [9]. Therefore, UA may play an endogenous danger role in cardiovascular disease. Many studies have also demonstrated that serum level of UA is associated with cardiovascular diseases [10].

Compared with late-onset coronary heart disease, early-onset coronary heart disease (EOCAD) has particular components of etiology, including family heredity, lipid metabolism, gender composition, and risk factors [11–13]. For instance, our previous study also found that the *MTHFR* gene C677T polymorphism was associated with risk of early-onset myocardial infarction (MI) but not in late-onset MI in Caucasians [14]. Many previous studies have suggested that serum UA is an important risk factor for coronary artery disease (CAD). However, we have found few studies considering the relationship between the serum levels of UA and risk of EOCAD. This study is being planned to investigate whether there is an association between serum UA levels and the presence and severity of EOCAD.

## 2. Materials and Methods

### 2.1. Study Subjects

This study was a hospital-based case-control study conducted in patients attending The Affiliated Hospital of Qingdao University from January 2013 to June 2017. The 1093 EOCAD patients were aged ≤50 years in their first onset of symptoms and hospitalization for coronary angiography. All patients fulfilled the criteria of stable chest pain and/or signs of myocardial ischemia on exercise electrocardiography. EOCAD was assessed by review of patients' angiograms by their treating cardiologists. Patients having history of chronic infection diseases, abnormal kidney function, pregnancy, and neoplastic disease and patients on treatment with drugs (diuretics, allopurinol) were excluded from the study. Age- and sex-matched apparently healthy people (*n* = 1117), free from any signs or symptoms of cardiovascular disease, served as control. They were recruited from a geographic background similar to that of the patients and came from community samples or hospital employees. Written informed consent was obtained from each patient included in the study. The study protocol conforms to the ethical guidelines of the 1975 Declaration of Helsinki and was conducted in accordance with the guidelines set by the Ethics Committee of The Affiliated Hospital of Qingdao University.

### 2.2. Anthropometric and Clinical Parameters

A full physical examination of all the subjects was carried out, and data involving smoking and drinking habits, body mass index (BMI), age at onset of CAD symptoms, MI, hypertension, diabetes mellitus, and dyslipidemia were recorded. Height and weight were recorded to the nearest 0.5 cm and 0.1 kg, respectively. The BMI was calculated by using the formula: body weight in kilograms divided by height in meters squared. Systolic and diastolic blood pressure was measured twice at an interval of 30 min using the automated oscillometric device. An average value of the two readings was considered. Diagnosis of hypertension was based on the presence of elevated systolic (≥140 mmHg) and/or diastolic (≥90 mmHg) blood pressure. Diabetes mellitus (DM) was diagnosed when the subject casual fasting glucose level ≥ 7.8 mmol/l, ≥11.1 mmol/l at 2 h after an oral glucose challenge or both.

### 2.3. Evaluation of CAD Severity

Coronary angiography was used to identify the number of diseased vessels in MI patients. Four major coronary artery branches (left main, left anterior descending, left circumflex, and right coronary artery) were evaluated, and a luminal stenosis degree of 50% or more was defined as a significant lesion. Patients were defined as having single, double, or triple branch involvement if they had one, two, and three or more branches involved, respectively.

The severity of CAD was calculated for each patient by the Gensini score system. The Gensini score is a point scale based upon the number of stenotic coronary artery segments, including the degree of luminal narrowing (the score was from 1 to 32) and the localization of the stenosis (the score was from 0.5 to 5). Thus, the Gensini score is calculated as a sum of stenosis score and functional significance score calculated for each segment of the coronary artery tree.

### 2.4. Biochemical Measurements

Blood samples were drawn from all participants after an overnight fasting of at least eight hours. Serum levels of fasting blood glucose (FBG), triglyceride (TG), total cholesterol (TC), low-density lipoprotein cholesterol (LDL-C), high-density lipoprotein cholesterol (HDL-C) and serum creatinine (SCr), Lipoprotein (a) (Lp (a)), and uric acid (UA) were determined by using the automatic biochemistry analyzer (Hitachi HCP-7600, Japan).

### 2.5. Statistical Analysis

All data were analyzed with SPSS statistical software (version 13.0; SPSS Inc., Chicago, Illinois, USA). Values are means ± standard deviation (SD) if not otherwise specified. The distribution of categorical variables was expressed as frequencies and percentages and the comparisons calculated by using chi-square test or Fisher exact test, as appropriate. Comparisons between groups for study variables were done using the unpaired Student's *t*-test or one-way ANOVA for normally distributed parameters. Spearman correlation coefficients were used to discern interrelationships. Logistic regression was used to test the interactive effects of other variables on the observed association between serum UA and EOCAD. All tests were two-sided, and throughout, *P* < 0.05 was considered statistically significant.

## 3. Results

### 3.1. Baseline Characteristics

A total of 1093 EOCAD patients and 1117 controls matched for age and gender were enrolled into the study. The demographic and clinical characteristics of the study population are shown in Table 1.

**Figure 1:**
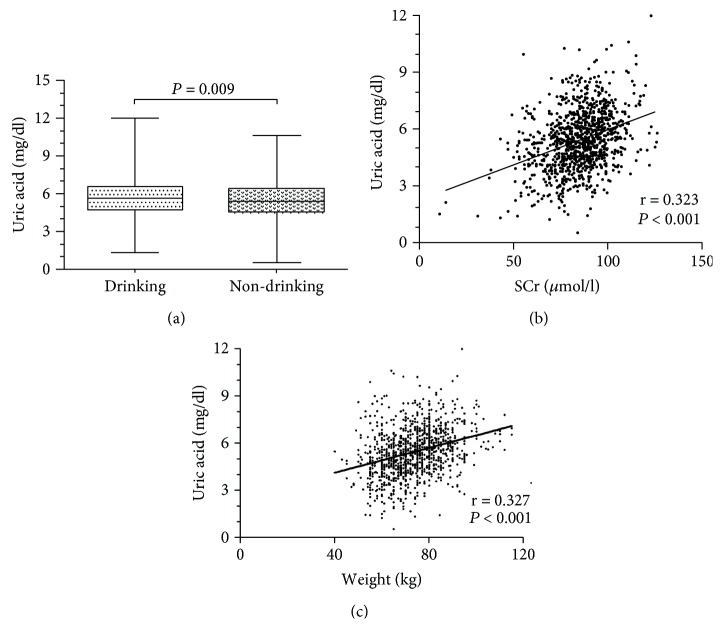
The influence factors of serum uric acid levels in EOCAD patients. (a) Male controls with drinking status (5.713 ± 1.505 mg/dl) had significantly higher serum uric acid levels than male controls without drinking status (5.463 ± 1.483 mg/dl, unpaired *t*-test, *P* = 0.009). (b) Correlation between serum uric acid and SCr in EOCAD patients (*r* = 0.323, *P* < 0.001). (c) Correlation between serum uric acid and weight in controls (*r* = 0.327, *P* < 0.001).

The EOCAD patients had higher values of BMI, FBG, TG, LDL-C, and Lp (a), whereas they displayed lower concentrations of HDL-C. Specifically, both groups had similar gender composition, age, serum TC levels, and SCr levels. In addition, the patients had higher smoking and drinking rate compared with controls. 633 patients were diagnosed as MI in the EOCAD group, including 326 patients with 1-vessel disease, 199 patients with 2-vessel disease, and 108 patients with 3-vessel disease. The drinking status could significantly increase the serum UA levels in the male control group (5.713 ± 1.505 mg/dl vs. 5.463 ± 1.483 mg/dl, *P* < 0.01, Figure 1(a)) but not in the male EOCAD patient group (5.966 ± 1.464 mg/dl vs. 5.981 ± 1.393 mg/dl, *P* > 0.05). The smoking status has no effect on the serum UA levels in male control (5.644 ± 1.453 mg/dl vs. 5.503 ± 1.522 mg/dl, *P* > 0.05) and EOCAD groups (6.002 ± 1.502 mg/dl vs. 5.931 ± 1.372 mg/dl, *P* > 0.05). In addition, we found that serum UA levels were weakly correlated with SCr (*r* = 0.247, *P* < 0.000 in the EOCAD group and *r* = 0.323, *P* < 0.001 in the control group, Figure 1(b)). Serum UA levels were positively correlated with weight (*r* = 0.327, *P* < 0.001, Figure 1(c)) in the control group.

### 3.2. The Relationship between Serum UA Levels and Risk of EOCAD

**Figure 2:**
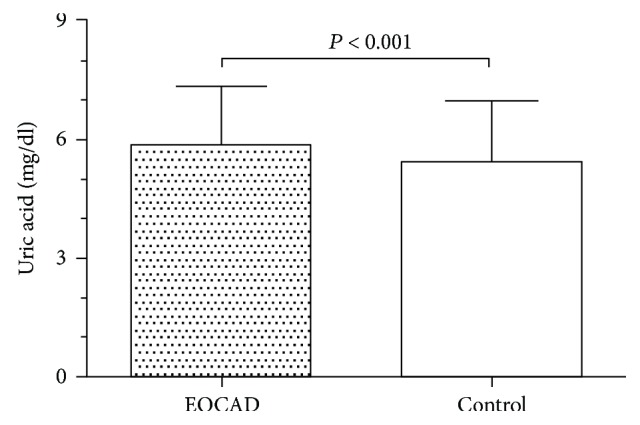
Serum uric acid levels in EOCAD and control groups. Serum uric acid levels in EOCAD patients (5.843 ± 1.479 mg/dl) were significantly increased when compared with that in healthy controls (5.433 ± 1.529 mg/dl, unpaired *t*-test, *P* < 0.001).

**Figure 3:**
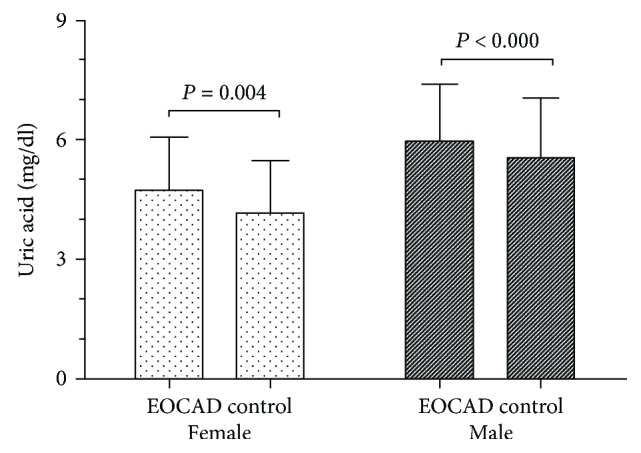
Comparing serum uric acid levels in EOCAD and control group by gender. Serum uric acid levels in the male and female group. Serum uric acid levels were significantly higher in the female EOCAD group (4.718 ± 1.343 mg/dl) than in the female control group (4.178 ± 1.311 mg/dl, unpaired *t-* test, *P* = 0.004). The same relationship was also detected in male EOCAD (5.965 ± 1.442 mg/dl) and male control group (5.560 ± 1.492 mg/dl, unpaired *t-* test, *P* < 0.001).

To examine the relationship between serum UA levels and risk of EOCAD, serum UA levels were measured in all 2210 subjects. The mean serum level of serum UA was 5.843 ± 1.479 mg/dl in EOCAD patients and 5.433 ± 1.529 mg/dl in controls. Serum UA levels were significantly higher in the EOCAD group than those in the control group (unpaired *t*-test, *P* < 0.001, Figure 2). Serum UA levels were significantly higher in the female EOCAD group (4.718 ± 1.343 mg/dl) than those in the female control group (4.178 ± 1.311 mg/dl, unpaired *t*-test, *P* < 0.01, Figure 3). The same relationship was also detected in the male EOCAD (5.965 ± 1.442 mg/dl) and male control group (5.560 ± 1.492 mg/dl, unpaired *t*-test, *P* < 0.001, Figure 3).

By logistic regression analysis, after adjusting for age, gender, BMI, diabetes, hypertension, smoking status, and drinking status, the association between UA and EOCAD was detected (OR = 1.150, 95% CI: 1.075–1.229; *P* < 0.001). After adjustment for Glu, LDL-C, SCr, TG, TC, HDL-C, and Lp (a), UA remained as a significant factor that correlated with EOCAD (OR = 1.139, 95% CI: 1.065–1.218; *P* < 0.001). After further adjustment for all these factors, UA remained as an independent risk factor for EOCAD (OR = 1.100, 95% CI: 1.022–1.185; *P* = 0.011) (Table 2).

### 3.3. The Relationship between Serum UA Levels and Severity of EOCAD

The serum UA levels in EOCAD patients with stable angina, unstable angina, and MI were 5.554 ± 1.419 mg/dl (*n* = 101), 5.886 ± 1.505 mg/dl (*n* = 344), and 5.880 ± 1.479 mg/dl (*n* = 633), respectively. The serum UA levels in patients with stable angina were significantly lower than those in patients with unstable angina and MI (one-way ANOVA, *P*_SAP vs.MI_ < 0.05, and *P*_SAP vs.UAP_ < 0.05, Table 3).

When all EOCAD patients with MI were divided into 3 groups according to the number of diseased vessels, serum UA levels were 5.783 ± 1.411 mg/dl (*n* = 326), 5.799 ± 1.503 mg/dl (*n* = 199), and 6.322 ± 1.554 mgl/dl (*n* = 108) in MI patients with 1–3-vessel disease. We detected significant difference in the three groups (one-way ANOVA, *P* < 0.01). Levels of serum UA in the group with 3-vessel disease were significantly higher than those in groups with 1-vessel disease and 2-vessel disease (one-way ANOVA, *P*_1 vs.3_ < 0.01 and *P*_2 vs.3_ < 0.01, Table 3).

Hyperuricemia was defined as serum UA ≥ 7 mg/dl in men or ≥6 mg/dl in women. The Gensini score was 56.33 ± 45.80 in male EOCAD patients with hyperuricemia (*n* = 222) and 48.25 ± 37.23 in male EOCAD patients without hyperuricemia (*n* = 764, unpaired *t*-test, *P* < 0.05, Table 4). In female EOCAD patients, the Gensini score was 71.77 ± 64.33 in patient with hyperuricemia (*n* = 82) and only 26.22 ± 22.50 in female EOCAD without hyperuricemia (*n* = 24, unpaired *t*-test, *P* < 0.05, Table 4).

## 4. Discussion

The present study demonstrated that (1) the serum UA levels were significantly elevated in EOCAD patients, and the serum UA was an independent risk factor for EOCAD. (2) Serum UA levels were associated with disease severity. (3) The serum UA levels were influenced with drinking status and were positively correlated with SCr and weight.

UA is a final product of purine metabolism, formed from the breakdown of adenosine and guanine. The transformation of hypoxanthine into xanthine and of the latter into UA is a biochemical chain that leads to UA generation. The processes are catalyzed by the enzyme xanthine oxidase [15]. UA is degraded by the urate oxidase, to allantoin, which is freely excreted into the urine [16]. UA is completely filtered through the glomerulus, completely reabsorbed in the proximal tubule, secreted, and finally, reabsorbed [17]. Normal serum levels of UA are generally 6.5 to 7 mg/dl for men and 6 to 6.5 mg/dl for women. Elevated serum UA is the result of overproduction or under excretion.

The positive association between serum UA and cardiovascular disease was recognized for 60 years. A number of epidemiologic studies have reported a relation between serum UA levels and a wide variety of cardiovascular conditions, including hypertension [18], metabolic syndrome [19], CAD [20], cerebrovascular disease [21], vascular dementia [21], and preeclampsia [22]. In the process of UA production, oxidants generated via xanthine oxidase may impair nitric oxide synthesis and availability [23]. UA could induce the proliferation and proinflammation of vascular smooth muscle cells [24]. Studies show that UA could induce CRP expression in endothelial cells, and this finding may provide further direct evidence for both proinflammatory and proatherogenic effects of UA [25]. Hyperuricemia is a frequent finding in insulin-resistant states and plays an important role in dysregulation of glucose uptake [26]. All these damage effects of UA were associated with endothelial dysfunction and finally lead to cardiovascular disease.

To compare with late-onset CAD, EOCAD may have different pathogenic mechanisms. Numerous studies have discovered so many biomarkers on EOCAD, including genes, proteins, and other bio-macromolecules [27, 28]. Our previous studies found that the *MTHFR* gene C677T polymorphism was associated with the risk of early-onset MI in Caucasians (OR = 1.275, 95% CI: 1.077–1.509) [14], and serum ADMA levels were associated with the presence and severity of EOCAD [29]. To our knowledge, only two studies explored the association between serum UA and risk of EOCAD in recent years. Otherwise, the debate as to whether UA plays an independent causal role in the development of CAD has intensified recently with many reviews and editorials providing differing views and recent studies providing conflicting results [30]. Zand et al.'s study only involved 245 EOCAD patients and 228 normal coronary subjects. They found that UA was significantly related to the presence of EOCAD, but it is not an independent risk factor for EOCAD [31]. Dai and coworkers demonstrated that higher serum UA level might play an important role in the severity of EOCAD in 786 patients. Because of the lack of the control group, the study could not discuss whether serum UA was an independent risk factor for EOCAD [32]. In our present study, we evaluate the association between serum UA levels and risk of EOCAD in 2210 participants (1093 patients and 1117 controls). The positive results were detected. Binary logistic regression clearly demonstrated that the serum UA was an independent risk factor for EOCAD. We also found the serum UA levels associated with the disease severity. Levels of serum uric acid in the MI group with 3-vessel disease were significantly higher than those in groups with 1-vessel disease and 2-vessel disease; the serum UA levels in patients with stable angina patients were significantly lower than in those with unstable angina and MI, and the Gensini score in EOCAD patients with hyperuricemia is higher than that in EOCAD patients without hyperuricemia. In addition, we also detected that the serum UA levels were influenced by drinking status and were positively correlated with SCr and weight.

The current study should be considered as a preliminary report which has some limitations. First, a control group comprised age- and sex-matched individuals without any signs or symptoms of CAD and having a normal result of routine blood tests. We cannot exclude that since coronary angiography has not been performed in all the controls. Second, the findings are based on cross-sectional data and only measured serum UA at first hospital admission. A temporal association between serum UA and development of EOCAD cannot be inferred with certainty.

## 5. Conclusions

In conclusion, we observed that increased serum UA levels were associated with EOCAD and first indicated that serum UA is an independent risk factor for the disease. We also demonstrated that the serum UA levels were associated with disease severity, affected with drinking status, and were positively correlated with SCr and weight. The findings may be useful in understanding the mechanism and predicting progression of patients with EOCAD and may contribute to the further prevention of the disease.

## Figures and Tables

**Table 1:** Demographic and clinical characteristics of EOCAD patients and controls.

Variable	EOCAD (*n* = 1093)	Control (*n* = 1117)	*P* value
Gender, male *n* (%)^#^	986 (90.21)	1015 (90.86)	0.597
Age, years^∗^	43.90 ± 4.61	43.83 ± 4.52	0.693
BMI (kg/m^2^)^∗^	26.78 ± 3.61	24.75 ± 3.38	<0.001
Hypertension, *n* (%)^#^	314 (28.73)	305 (27.31)	0.578
Diabetes, *n* (%)^#^	214 (19.58)	46 (4.12)	<0.001
Smoking, *n* (%)^#^	533 (48.76)	446 (41.72)	<0.001
Drinking, *n* (%)^#^	677 (61.94)	415 (39.92)	<0.001
FBG, mmol/l^∗^	6.06 ± 2.57	5.43 ± 1.63	<0.001
TG, mmol/l^∗^	2.15 ± 1.84	1.45 ± 1.13	<0.001
TC, mmol/l^∗^	4.54 ± 1.29	4.52 ± 1.96	0.635
HDL-C, mmol/l^∗^	1.08 ± 0.27	1.27 ± 0.33	<0.001
LDL-C, mmol/l^∗^	2.62 ± 0.99	2.51 ± 0.74	0.004
Lp(a), mmol/l^∗^	287.89 ± 331.77	205.65 ± 218.89	<0.001
SCr, *μ*mol/l^∗^	73.33 ± 15.86	75.14 ± 13.81	0.139
UA, *μ*mol/l^∗^	5.843 ± 1.479	5.433 ± 1.529	<0.001
Male, *μ*mol/l^∗^	5.965 ± 1.442	5.560 ± 1.492	<0.001
Female, *μ*mol/l^∗^	4.718 ± 1.343	4.178 ± 1.311	0.004
Myocardial infarction, *n* (%)	633 (57.91)	—	—
1-vessel disease, *n* (%)	326 (51.50)	—	—
2-vessel disease, *n* (%)	199 (31.44)	—	—
3-vessel disease, *n* (%)	108 (17.06)	—	—

EOCAD: early-onset coronary artery disease; BMI: body mass index; FBG: fasting blood glucose; TG: triglyceride; TC: total cholesterol; HDL-C: high-density lipoprotein cholesterol; LDL-C: low-density lipoprotein cholesterol; Lp (a): lipoprotein (a); SCr: serum creatinine; UA: uric acid. ^∗^Continuous variables are expressed as mean ± SD. The *P* value of the continuous variables was calculated by the unpaired *t*-test. ^#^Categorical variables are expressed as percentages. The *P* value of the categorical variables was calculated by *χ*^2^ test.

**Table 2:** Associations between serum uric acid and risk of EOCAD.

	OR 95% CI	*P* value
Model 1: crude, no adjustment	—	<0.001
Model 2: adjusting for age, gender, BMI, diabetes, hypertension, smoking status, and drinking status	1.150 (1.075–1.229)	<0.001
Model 3: adjusting for FBG, LDL-C, SCr, TG, TC, HDL-C, and Lp(a)	1.139 (1.065–1.218)	<0.001
Model 4: adjusting for all these factors	1.100 (1.022–1.185)	0.011

BMI: body mass index; FBG: fasting blood glucose; TG: triglyceride; TC: total cholesterol; HDL-C: high-density lipoprotein cholesterol; LDL-C: low-density lipoprotein cholesterol; SCr: serum creatinine; Lp (a): lipoprotein (a).

**Table 3:** The serum UA levels and severity of EOCAD.

	*n*	UA (mg/dl)	*P* value^∗^
*Subtype of EOCAD*			
SAP	101	5.554 ± 1.419	*P*_SAP vs.MI_ < 0.05
UAP	344	5.886 ± 1.505	*P*_UAP vs.MI_ > 0.05
MI	633	5.880 ± 1.479	*P*_SAP vs.UAP_ < 0.05
*Number of diseased vessels in MI*			
1-vessel disease	326	5.783 ± 1.411	*P*_1 vs.2_ > 0.05
2-vessel disease	199	5.799 ± 1.503	*P*_1 vs.3_ < 0.01
3-vessel disease	108	6.322 ± 1.554	*P*_2 vs.3_ < 0.01

EOCAD: early-onset coronary artery disease; UA: uric acid; SAP: stable angina; UAP: unstable angina; MI: myocardial infarction. ^∗^The *P* value was calculated by one-way ANOVA.

**Table 4:** Association of serum uric acid categories and Gensini score in male and female EOCAD patients.

Variable	Male EOCAD patients			Female EOCAD patients		
	Normouricemia (<7.0 mg/dl)	Hyperuricemia (≥7.0 mg/dl)	*P* value^∗^	Normouricemia (<6.0 mg/dl)	Hyperuricemia (≥6.0 mg/dl)	*P* value^∗^
Number, *n*	764	222	*—*	82	24	*—*
Age, years	43.74 ± 4.55	43.40 ± 1.99	0.333	46.18 ± 3.59	45.18 ± 3.06	0.379
BMI	26.59 ± 3.54	27.86 ± 3.65	<0.001	25.94 ± 3.52	25.98 ± 4.07	0.967
FBG, mmol/l	6.07 ± 2.53	5.72 ± 2.06	0.067	6.59 ± 3.29	8.04 ± 5.11	0.197
TG, mmol/l	2.01 ± 1.53	2.78 ± 2.49	<0.000	1.83 ± 2.10	1.63 ± 0.50	0.744
TC, mmol/l	4.52 ± 1.27	4.65 ± 1.38	0.170	4.45 ± 1.17	4.40 ± 2.36	0.953
HDL-C, mmol/l	1.07 ± 0.26	1.03 ± 0.25	0.057	1.21 ± 0.29	1.04 ± 0.23	0.042
LDL-C, mmol/l	2.64 ± 0.97	2.59 ± 1.02	0.586	2.45 ± 0.86	2.69 ± 1.93	0.686
Lp(a), mmol/l	289.96 ± 344.69	262.95 ± 281.29	0.286	331.47 ± 345.69	257.07 ± 193.18	0.486
SCr, *μ*mol/l	85.71 ± 14.80	92.55 ± 15.89	<0.001	69.65 ± 14.31	77.48 ± 13.46	0.017
Gensini score	48.25 ± 37.23	56.33 ± 45.80	**0.017**	26.22 ± 22.50	71.77 ± 64.33	**0.044**

EOCAD: early-onset coronary artery disease; BMI: body mass index; FBG: fasting blood glucose; TG: triglyceride; TC: total cholesterol; HDL-C: high-density lipoprotein cholesterol; LDL-C: low-density lipoprotein cholesterol; Lp (a): lipoprotein (a); SCr: serum creatinine; UA: uric acid. ^∗^Continuous variables are expressed as mean ± SD. The *P* value of the continuous variables was calculated by unpaired *t*-test.

## Data Availability

The datasets used and/or analyzed during the current study are available from the corresponding author on reasonable request.
